# Mapping the cognitive connectome of men and women, a Graph Theory study across three cohorts

**DOI:** 10.1002/alz70857_103196

**Published:** 2025-12-25

**Authors:** Daphne Gasparre, Lídia Mulet‐Pons, Annegret Habich, Roraima Yánez‐Pérez, Jose Barroso, Lídia Vaqué‐Alcázar, David Bartrés‐Faz, Paolo Taurisano, Daniel Ferreira

**Affiliations:** ^1^ Department of Translational Biomedicine and Neuroscience “DiBraiN”, University of Bari Aldo Moro, Bari, Italy; ^2^ Division of Clinical Geriatrics, Center for Alzheimer Research, Department of Neurobiology, Care Sciences and Society, Karolinska Institutet, Stockholm, Sweden; ^3^ Department of Medicine, Faculty of Medicine and Health Sciences, Institute of Neurosciences, University of Barcelona, Barcelona, 08036, Spain; ^4^ University of La Laguna, San Cristóbal de La Laguna, Spain; ^5^ Department of Medicine, Faculty of Medicine and Health Sciences, Institute of Neurosciences, University of Barcelona, Barcelona, Spain; ^6^ Sant Pau Memory Unit, Department of Neurology, Hospital de la Santa Creu i Sant Pau, Biomedical Research Institute Sant Pau, Universitat Autònoma de Barcelona, Barcelona, Spain

## Abstract

**Background:**

Cognitive processes are crucial for daily functioning and their impairment significantly impacts quality of life. Age and education are known to influence cognition, but the role of sex is less clear. While univariate studies report mixed results on the effect of sex on cognition, graph theory offers a comprehensive approach to analyzing complex associations among cognitive measures. This study examined sex differences in cognition using graph theory and compared the traditional univariate approach with graph theory analysis.

**Method:**

We compared sex differences in cognition in three cohorts of cognitively healthy adults: 334 participants from the GENIC dataset, 3703 from NACC, and 222 from ADNI. Cognitive variables adjusted for age and education were used to build cognitive connectomes using Spearman correlation coefficients. Differences in cognitive performance were evaluated using ANCOVAs, correcting for age and education (univariate approach). Global and nodal graph metrics were examined using graph theory (multivariate approach). Graph theory was also employed for modular analysis. All analyses were conducted in MATLAB.

**Result:**

The univariate approach showed sex differences in memory and learning, language, and complex attention with small effect sizes. Graph analyses showed no differences in global features of the cognitive connectomes between men and women. On the contrary, nodal analyses identified sex differences in the investigated cognitive domains, with more differences in memory and learning, language, and executive functions. Modular analysis showed rather similar modules between women and men with nuanced differences in language, attention and executive functions.

**Conclusion:**

Graph theory provides an innovative approach to studying sex differences in cognitive performance. The cognitive connectomes of men and women do not differ in global features, while there are nuanced differences in nodal and modular features of language, executive and attention networks. This finding suggests that sex differences could be related to how men and women approach specific cognitive tasks rather than overt differences in cognitive ability. These findings may have potential implications for targeted interventions to support cognitive functioning in men and women.

